# A case of papuloerythroderma of Ofuji treated with upadacitinib

**DOI:** 10.1016/j.jdcr.2024.11.021

**Published:** 2024-11-29

**Authors:** Kexin Lei, Jiahui Zhao

**Affiliations:** Department of Dermatology and Venereology, Peking University First Hospital, National Clinical Research Center for Skin and Immune Diseases, Beijing Key Laboratory of Molecular Diagnosis on Dermatoses, National Medical Products Administration Key Laboratory for Quality Control and Evaluation of Cosmetics, Beijing, China

**Keywords:** JAK inhibitor, papuloerythroderma of Ofuji, upadacitinib

## Introduction

Papuloerythroderma of Ofuji (PEO) is a rare and distinctive dermatologic condition characterized by the appearance of polygonal, flat-topped erythematous-brown papules that coalesce into extensive sheets, covering the skin surface but sparing skinfolds—a phenomenon known as the “deck-chair sign.”^1^ This clinical presentation was described by Ofuji et al^2^ in Japan in 1984, marking its recognition as a distinct entity.

Although the pathogenesis of PEO remains elusive, existing literature suggests plausible associations with malignancies, medications, infections, and atopic diseases.^3^ PEO primarily affects older men and often presents with symptoms such as itching and laboratories abnormalities such as peripheral eosinophilia and elevated levels of IgE.^4^ Despite being recognized for decades, treatment options for PEO remain limited. In a departure from conventional approaches, we present here, for the first time to our knowledge, upadacitinib, an oral selective Janus kinase 1 inhibitor, as a potential treatment strategy for PEO. This innovative therapeutic approach represents a substantial advancement in addressing the challenges posed by this intriguing dermatologic condition, warranting further investigation and to enhance patient care.

## Case report

**Fig 1:**
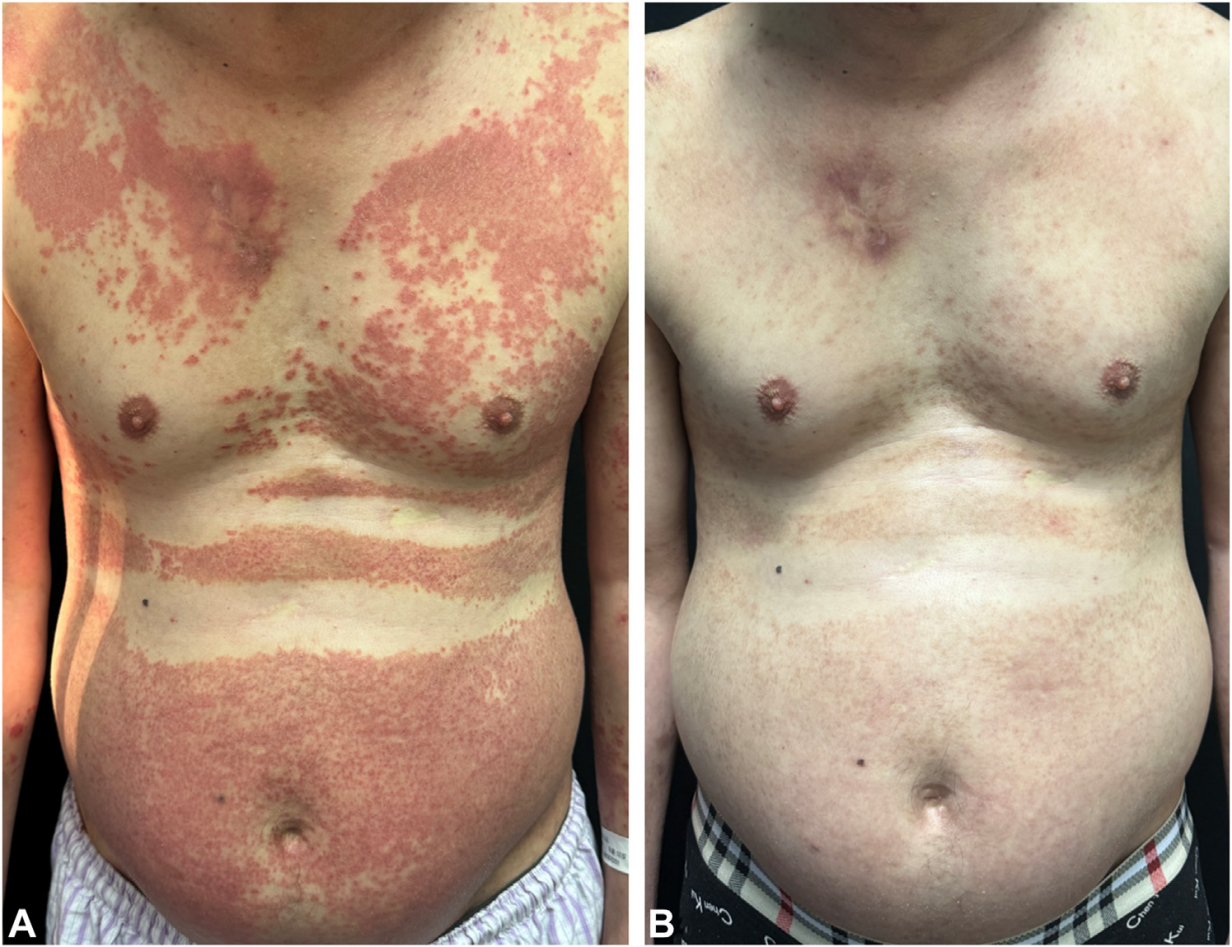
**A,** Red papules on the trunk progressing to erythroderma sparing the skinfolds (deck-chair sign). **B,** After 4 weeks of treatment, the eruption had significantly improved.

**Fig 2:**
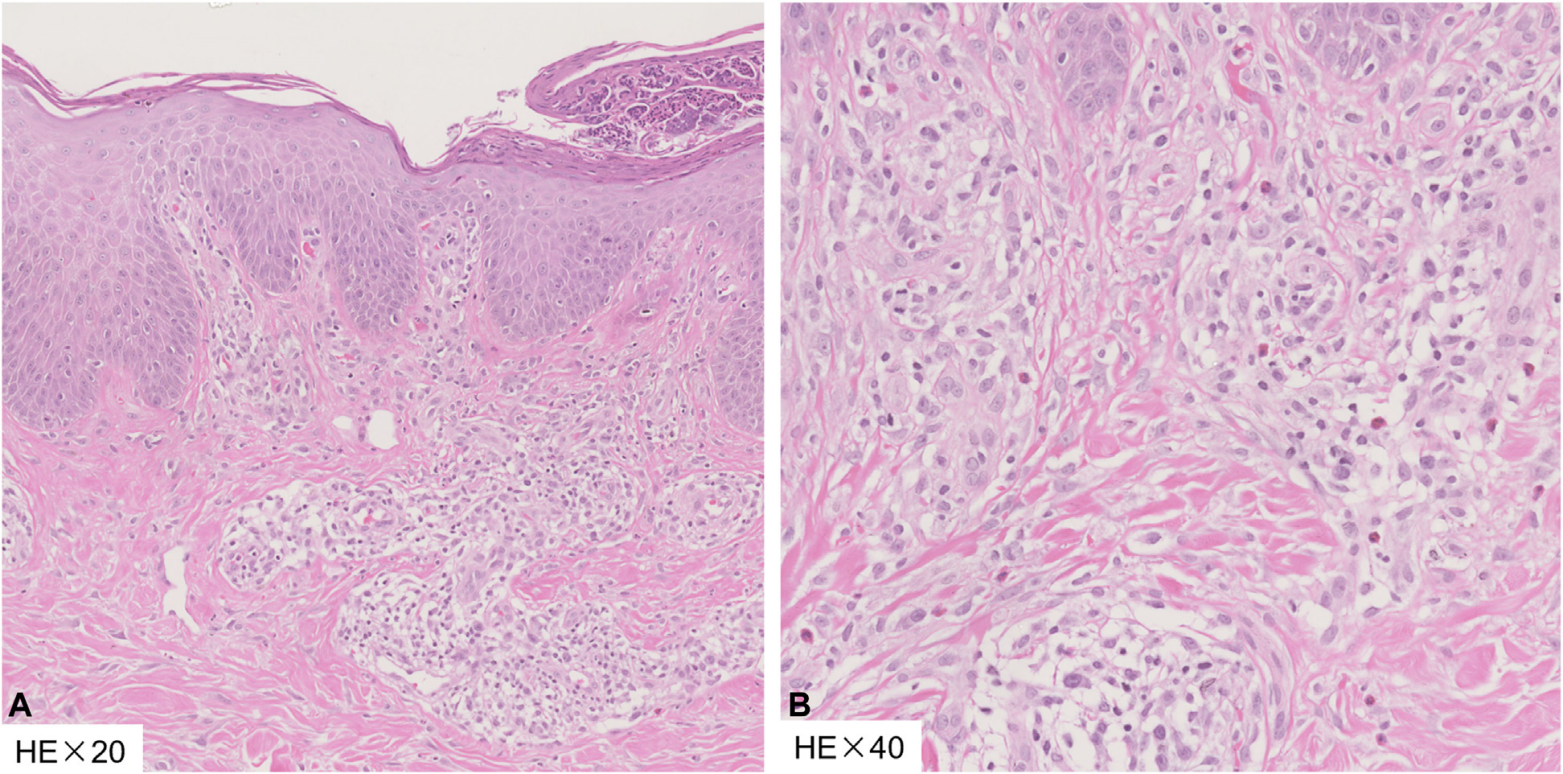
Skin biopsy demonstrating edema in the stratum spinosum and perivascular lymphocytic infiltration with multiple eosinophils, consistent with subacute dermatitis (**A** and **B,** Hematoxylin-eosin stain; original magnifications: **A,** ×20; **B,** ×40.).

A 52-year-old man presented to our dermatology clinic with a 7-month history of pruritic, red papules scattered across his trunk, which had gradually progressed to erythroderma, notably sparing the skinfolds (Fig 1, *A*). Comprehensive patient histories, including information on cancer, infections, and drug usage, were acquired. Pulmonary computed tomography imaging and infectious tests for hepatitis B virus, hepatitis C virus, HIV, and treponema pallidum antibodies were carried out. However, no underlying causes were identified. Laboratory investigations revealed eosinophilia (0.84 × 10^9^/L) and elevated serum lactate dehydrogenase levels (303 IU/L), whereas total IgE levels remained within normal limits (99.6 kU/L). Histology exhibited mild thickening of the stratum corneum, acanthosis with spongiosis, and perivascular dermatitis with eosinophils, consistent with spongiotic dermatitis (Fig 2). Despite prior treatment attempts with topical steroids and traditional Chinese medicine yielding limited improvement, the patient’s condition persisted. Because of the numerous side effects associated with systemic glucocorticoid—such as hypertension, weight gain, and osteoporosis—the patient declined glucocorticoid therapy. Consequently, a therapeutic regimen comprising upadacitinib (30 mg once daily) alongside antihistamines was initiated. After 4 weeks of treatment, the eruption and pruritus had nearly resolved (Fig 1, *B*). After 6 months of treatment, the patient’s upadacitinib dosage was tapered to 15 mg once daily. Subsequently, the patient was diligently monitored over an 8-month period. Notably, the rash is currently well-controlled, with no indication of recurrence during this follow-up period.

## Discussion

In this case, notable improvement in cutaneous lesions was observed within 4 months of initiating treatment with upadacitinib. Additionally, there was a marked reduction in blood eosinophil counts posttreatment (0.07 × 10^9^/L).

Traditionally, primary management of PEO has centered around the use of topical or systemic corticosteroids.^5^ Systemic therapies have included photochemotherapy, methotrexate, azathioprine, etretinate, and cyclosporine. PEO onset may be associated with interleukins 4 and 13 pathways, as well as activation of T helper 2 and T helper 22 cells.^4^ Interestingly, a case report has shown that therapeutic response using interleukin 4 receptor alpha monoclonal antibody dupilumab.^5^ Mechanistically, Janus kinase inhibitor may also prove beneficial, as demonstrated in our successful treatment of this case. Controlled trials would be beneficial in elucidating the role Janus kinase inhibitors will play in the treatment of PEO.

Given the rarity of PEO and the absence of a widely accepted treatment approach, our case emphasizes the potential effectiveness of upadacitinib. This case report not only show cases the versatility of upadacitinib in addressing challenging skin conditions but also underscores the need for further investigation and confirmation of its therapeutic benefits in larger PEO patient populations.

## Conflicts of interest

None disclosed.
